# The role of interleukin-12 in the heavy metal-elicited immunomodulation: relevance of various evaluation methods

**DOI:** 10.1186/1745-6673-3-25

**Published:** 2008-11-06

**Authors:** Nasr YA Hemdan

**Affiliations:** 1 Fraunhofer Institute for Cell Therapy and Immunology (IZI), Leipzig, Germany; 2 Institute of Clinical Immunology and Transfusion Medicine – IKIT, Faculty of Medicine, University of Leipzig, Germany; 3 Department of Zoology, Faculty of Science, University of Alexandria, Egypt

## Abstract

**Background:**

Increasing evidence exists that heavy metals modulate T helper cell (Th) responses and thereby elicit various pathological manifestation. Interleukin (IL)-12, a crucial innate cytokine, was found to be regulated by such xenobiotic agents. This study aimed at testing whether IL-12 profiles may be indicative of heavy metals-induced immunomodulation.

**Methods:**

Human immunocompetent cells, activated either by monoclonal antibodies or heat-killed *Salmonella enterica*, were cultured in the absence or presence of cadmium (Cd) acetate or mercuric (Hg) chloride. *In vivo *experiments were set up where BALB/c mice were exposed to sub-lethal doses of Cd or Hg salts for 3 or 5 weeks. Cytotoxicity was assessed by MTT-reduction assay. Modulation of cytokine profiles was evaluated by enzyme-linked immunosorbent assay (ELISA), cytometric bead-based array (CBA) and real-time polymerase chain reaction (RT-PCR); the relevance of these methods of cytokine quantification was explored.

**Results:**

Modulation of IL-12 profiles in Cd- or Hg-exposed human PBMC was dose-dependent and significantly related to IFN-γ levels as well as to the Th1- or Th2-polarized responses. Similarly, skewing the Th1/Th2 ratios *in vivo *correlated significantly with up- or down-regulation of IL-12 levels in both cases of investigated metals.

**Conclusion:**

It can be inferred that: (i) IL-12 profiles alone may represent a relevant indicator of heavy metal-induced immune modulation; (ii) evaluating cytokine profiles by CBA is relevant and can adequately replace other methods such as ELISA and RT-PCR in basic research as well as in immune diagnostics; and (iii) targeting IL-12 in therapeutic approaches may be promising to modify Th1/Th2-associated immune disorders.

## Background

Human activities have led to global dispersion of heavy metals like cadmium and mercury into the environment [1-3], and hence heavy metal pollution has attained high visibility in the public arena and increasingly become a major scientific concern. Most of the early studies addressed the effects of relative higher doses that are not relevant to most populations. Despite intensive recent studies with regard to the immune system as a target of heavy metals, some inconsistencies have been evolved especially regarding the effects of low-dose exposure. The reported impairments ranged from minor insidious or transitional changes up to occasional death of the exposed subjects [1-8]. This variation in susceptibility has been attributed to genetic variability, variety of methodological regimes such as the metal form, duration of exposure, the dosage applied as well as the activity of the cells [9]. The biological indicator or the read-out system used to evaluate the changes elicited can not be ruled out.

Cytokines are very important agents to detect modulations of the immune system. Due to their sensitivity, they can be modulated at lower doses than other arms of the immune system [10]. Measuring cytokines and other soluble mediators involved in immune regulation has been the focus of researchers for over three decades. Several reports on the quantification of ever-growing number of analytes in a single reaction vessel have been emerged using fluorescent-labeled microspheres, the protein bead array (PBA)-based assays [11-17]. The importance of assessing cytokines is evidenced by the fact that they form the basis of a sophisticated cellular communication network for normal as well as modulated immune responses. Experiments using isolated human cells or cytokine gene knock-out mice have been proven to be useful for evaluating the regulation of immunocompetent cells in response to infection or following exposure to heavy metals. Central to these regulatory agents are CD4^+ ^T helper (Th) cells, that are known to differentiate into at least two subsets, Th1 and Th2, both in mice [18] and in humans [19]. These cells secrete different but overlapping sets of cytokines: the common precursor Th0 secrete cytokines including IL-2, IL-4, and IFN-γ; Th1 cells produce IL-2, IFN-γ, TNF-β, and low levels of IL-10 (only in human); and Th2 cells IL-4, IL-5, IL-9, IL-10, IL-13 [20-22] and IL-6 [23]. Differentiation of Th0 is believed to be a consequence of several cellular influences, such as the cytokine milieu. While differentiation into Th1 cells requires the presence of IL-12, Th2 cells response warrants the presence of IL-4 [24]. Moreover, both types cross-regulate each other in a variety of ways [20,24,25]. The dominance of one of these subsets results in either a predominantly cellular (Th1-mediated) or an antibody (Th2-mediated) response [9]. Therefore, the inclination of the Th1/Th2 balance indicated by cytokine profiles changes and/or cytokine-dependent regulatory pathways have been often considered to evaluate heavy metal-mediated immunomodulation and in risk assessment studies [26].

The recent years were fruitful with the definition of new Th subsets including the CXCR5^+ ^and CD4^+^CD25^+^T_reg _cells [27]. T_reg _cells includes, among others, constitutive CD4^+^CD25^+ ^FoxP3^+ ^T_reg _cells, Type 1 T regulatory cells (Tr1) and Th3 cells, characterized by production of high levels of IL-10 and TGF-β [28]. Most recently, studies of experimental autoimmune encephalomyelitis and adjuvant-induced arthritis have pointed to the importance of IL-17-producing (Th17) cells, [27]. The optimal recipe to differentiate into Th17 remains so far unclear; yet, together with TGF-β [29-31], a group of cytokines produced by LPS-activated DC, namely IL-1, IL-6, and TNF-α favor Th17 differentiation [32]. Other cytokines seem to share inducing or maintaining different Th cell subsets, e.g. IL-23, due to its p40 unit, just like IL-12, in addition to induction of Th1, is known to be important for the survival of Th17 cells [33].

Interleukin-12 is a 70-kDa heterodimeric (composed of covalently linked p35/p40) pro-inflammatory cytokine produced mostly by phagocytic cells and to some degree by B cells. It is considered to date the most critical factor for skewing the immune response towards a Th1 type, and thereby exerts a substantial stimulatory influence on host responses to intracellular pathogens [34-36]. However, there are clues that IL-12, in synergy with IL-4, supports the long-term proliferation and maturation of resting neonatal CD4^+ ^T cells into IFN-γ – or IL-4-producing cells, and transiently increases the production of both cytokines by human Th2-like cell clones [37]. The present study was conducted to examine the relationship between heavy metal-induced IL-12 profile modifications and the accompanying Th1/Th2-polarized responses of cultured human peripheral blood mononuclear cells (PBMC). To this end, two pathways of cell activation were adopted, either through monoclonal antibodies (mAb: anti-CD3/-CD28/-CD40) or heat-killed *Salmonella enterica *serovar Enteritidis (hkSE). Furthermore, the association of IL-12 with a skewed *in vivo *immune response was also investigated in BALB/c mice exposed to heavy metals in a pathogen-free environment. In order to test the metal effects at the protein as well as mRNA levels and to evaluate the relevance of various traditionally-used methods, cytokines were assessed by ELISA, cytometric bead-based array (CBA) and RT-PCR.

## Methods

### Preparation of cells

Cells used in this study were isolated from buffy coats of healthy blood donors from the Blood Bank of Leipzig University Clinic, Germany. The experiments were approved by the local authorities and informed consents of participating subjects were obtained. Ficoll Paque (Amersham Biosciences, Freiburg, Germany) density gradient centrifugation at 22°C and 400× g [38] was applied to separate PBMC. Cells were finally washed with isotonic phosphate-buffered saline (Invitrogen, Karlsruhe, Germany).

### Phenotypes of PBMC

Analysis of the human PBMC subsets was performed using surface marker staining and flow cytometry as previously described [26]. Briefly, sets of mAbs against surface antigens were used (BD Biosciences, Heidelberg, Germany): Simultest™ CD3/CD8, CD3/CD4, CD3/CD19, CD3/CD16CD56, Simultest™ Leucogate™ (CD45/CD14) and Simultest™ Control γ1/γ2a (IgG_1 _FITC/IgG_2a _PE). Lymphocytes were gated in the forward-side scatter plot and various cell subsets were estimated.

### Cell cultures

Isolated human PBMC were suspended in HybridoMed DIF 1000 medium (Biochrom, Berlin, Germany) containing 10 μg/mL gentamycin, 100 μg/mL streptomycin, 100 U/mL penicillin and 10% FCS (HyClone Laboratories, Logan, UT, USA) and incubated at 37°C/5% CO_2 _and finally cultured (1 × 10^6 ^cells/1 mL/well) in 48-well microtiter plates (Greiner Bio-one GmbH, Nürtingen, Germany). Cells were activated with agonistic CD3 (OKT3, mouse IgG1, Ortho Biotech, Bridgewater, NJ, USA), CD28 (clone CD28.2, mouse IgG1. Beckman-Coulter, Krefeld, Germany) and CD40 (clone B-B20, Trinova Biochem, Gießen, Germany) mAb, 100 ng/mL each, or with hkSE (1.25 × 10^5 ^CFU/mL; ade^-^, his^-^, SALMOVAC SE^®^, Impfstoffwerk Dessau-Tornau, Rosslau, Germany).

### Application of heavy metals

Cd acetate and Hg chloride (Sigma, Steinheim, Germany) were dissolved in de-ionized water (stock solution = 10 g/L). Immediately before application, serial dilutions were made using the same culture medium and added to the culture plates to constitute a final concentration of Cd or Hg ranged from 0.5 ng to 50 μg/mL. Control samples were established, where cells received only either mAb or hkSE.

### Cell vitality assay

Vitality response of human PBMC to mAb or hkSE was evaluated by 3-[4,5-dimethylthiazol-2yl]-2,5-diphenyltetrazoliumbromide (MTT)-reduction test as previously reported [26].

### Detection of cytokine release by ELISA

Following 24-hr incubation, cytokine levels were determined in culture media by commercially available ELISA kits for IL-1β, IL-10, IL-12p70, IL-4, IL-6, IFN-γ, and TNF-α (OptEIA™ Kits; BD Biosciences) with a lower detection limit of 4 pg/mL, as previously described [26,39].

### Animal model

Female wild-type BALB/c mice (9–12-week old, 23–27 g; purchased from Charles River, Sulzfeld, Germany), were allocated to this study. The animals were handled according to the animal protection laws of local authorities on the use of animals in research. Upon receipt from the vendor, mice were quarantined and acclimatized for 2 weeks prior to use. Animals were placed in filter-topped plastic cages (6–8 per cage) in exposure rooms with automatic 12:00-hr light 12:00-hr dark cycle, and allowed free access to food and water. Rooms were maintained at 22°C and 40–60% humidity.

Mice were assigned in groups of 12 mice and were injected intraperitoneally every third day with isotonic NaCl solution (controls) or with Cd or Hg (1.25 mg/kg body weight). Mice were monitored daily for food and water consumption and for signs of morbidity. Control mice were sacrificed at day 21 and other mice were sacrificed following 3 or 5 weeks of exposure to the heavy metal. At sacrifice, blood samples were collected and sera were separated by centrifuging the samples at 2,000 × g/37°C for 10 min.

### Cytokine analysis using cytometric bead array

Serum cytokine levels were determined using CBA. The procedure was carried out according to the manufacturer's instruction (CBA™, BD Biosciences, San Jose, CA), modified by Tarnok *et al*. [40] to measure the cytokines using 25 μL serum. Here, the test samples were further reduced to use 20 μL of 1:2 diluted sera. Briefly, 4 μL of each mouse capture bead suspension were mixed for each sample, and 20 μL of mixed beads were transferred to each assay tube. Standard dilutions or test samples were added to the appropriate tubes (20 μL/tube), PE detection reagent (20 μL) was added and the tubes were incubated for 2 hr in dark at RT. Samples were washed with 1 mL wash buffer and centrifuged at 200 × g for 5 min. Finally, test buffer (250 mL) was added, and samples were analyzed on FACSCalibur (BD Biosciences) using the supplied cytometer setup beads and the CellQuest™ Software.

### Evaluating Cytokines mRNA Expression

#### Isolation of RNA and digestion of genomic DNA

Following collection of organs, 1/4 spleen was transferred into 1 mL RNA *later*^® ^(Ambion, Germany), preserved overnight at 4°C and thereafter at -20°C. About 50 mg spleen was homogenized in 1 mL TriFast reagent (peqlab Biotechnologie, Erlangen, Germany) and RNA was separated according to the manufacturer's instruction. RNA probes were treated with DNA-free™ reagent (Ambion, Dresden, Germany) to eliminate genomic DNA.

#### Reverse transcription and purification of cDNA

Reverse transcription of RNA was conducted using AMV reverse transcriptase (Promega, Madison, USA), and cDNA probes were purified using QIAquick PCR Purification Kit (Qiagen) according to the manufacturer's instruction and were stored at -80°C.

#### Real-time PCR

Amplification of cDNA was performed on LightCycler using LightCycler-FastStart DNA Master^PLUS ^SYBR Green I^® ^(Roche, Mannheim, Germany). A 20-μL reaction mixture including 4 μL water, 2 μL primers, 9 μL DNA Master Mix and 5 μL (~0.2 μg) cDNA template was applied into the capillary and shortly centrifuged (3,000 × g). Reactions started by an initial activation step for 10 min at 95°C, and each following cycle started by a denaturation step for 15 s at 94°C. Specific cycle conditions were applied to validate the specificity for each primer pair. Products were controlled by SYBR Green dissociation curves, by agarose gel electrophoresis and via DNA-sequencing of the PCR products to ensure that only a single target-specific product of the appropriate length was amplified. GAPDH was chosen as a reference house-keeping gene as it showed amplification efficiency similar to those of other cytokine genes. The sequences of sense (s) and antisense (as) mouse primers were: (i) *GAPDH *(NM_008084): s: 5'-CCC ACT AAC ATC AAA TGG GG-3'; as: 5'-CCT TCC ACA ATG CCA AAG TT-3'; (ii) *IFN-γ *(NM_008337): s: 5'-AGC GGC TGA CTG AAC TCA GAT TGA AG-3', as: 5'-GTC ACA GTT TTC AGC TGT ATA GGG-3'; (iii) *IL-12p40 *(NM_008352): s: 5'-GGA AGC ACG GCA GCA GAA TA-3', as: 5'-AAC TTG AGG GAG AAG TAG GAA TGG-3' and (iv) *IL-4 *(NM_021283): s: 5'-TCA ACC CCC AGC TAG TTG TC-3' and as: 5'-TCT GTG GTG TTC TTC GTT GC-3'. The crossing point for each reaction was determined using the Second Derivative Maximum algorithm and the arithmetic baseline adjustment using the LightCycler software. REST^© ^software (downloaded from ) was used to estimate cytokine mRNA expression as well as the up- or down-regulation factor for each gene relative to controls and based on an efficiency corrected mathematical model and a pair-wise fixed reallocation randomization test [41].

### Statistical analysis

Experiments were carried out in triplicates. Data analysis was performed using Statistica 5.1 software (Statsoft, Hamburg, Germany). Variations among cytokine profiles of different donors were tested using a nonparametric ANOVA, the Kruskal-Wallis test, followed by Dunn's post-test. Wilcoxon's rank test for paired samples was used to analyze the differences between controls and heavy metal-treated cells in human PBMC cultures. The correlation between cytokine production and heavy metal doses a well as between cytokine pairs was analyzed using Spearman's rank correlation test. Unless otherwise indicated, significance was determined at p < 0.05.

## Results

### Distribution of different cell subsets

Isolated human PBMC were used in the *in vitro *studies. Of the total cells, the percentages of lymphocytes, monocytes, granulocytes as well as the lymphocyte subpopulations were in the normal range as previously reported [26].

### Exposure to heavy metals significantly modulates IL-12 profiles of human PBMC

**Figure 1:**
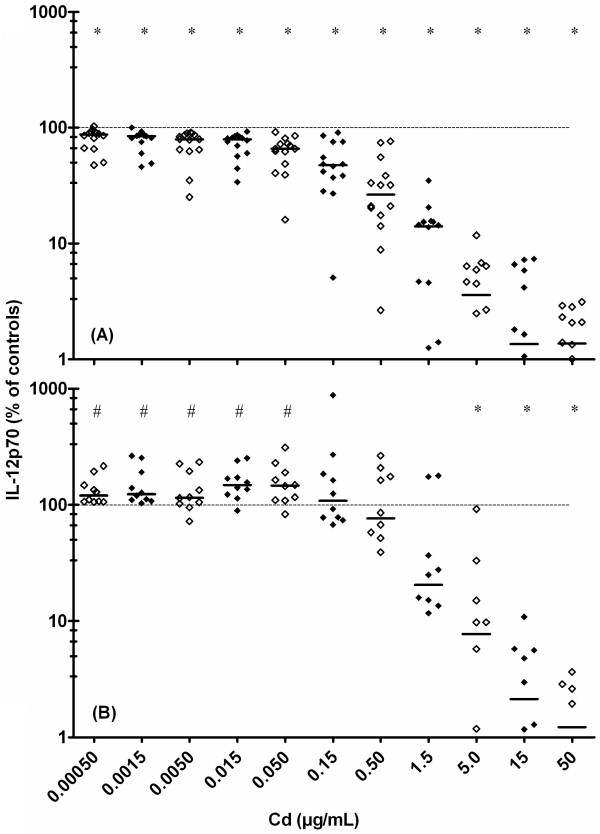
**Levels of IL-12p70 released by human PBMC exposed to Cd acetate for 24 hr**. Human PBMC were stimulated either by mAb (anti-CD3/-CD28/-CD40) (A) or heat-killed *Salmonella enterica *(hkSE) (B). Data represent the values of 14 and 10 samples. For each donor, the mean values of 3 replicates were used to estimate the percentage relative to control cells (assigned to 100%). The horizontal lines represent the medians. Symbols above each plot show whether, cytokine release was significantly stimulated (#) or suppressed (*) compared to controls (Wilcoxon's Rank Sum test for paired samples; p < 0.05).

Profiling IL-12 of mAb-stimulated PBMC revealed that 24-hr exposure to Cd significantly decreased IL-12p70 release (p < 0.01, Wilcoxon's test) at all tested doses from 0.5 ng/mL to 50 μg/mL (Fig. 1A); additional file 1. Values of IL-12p70 in the supernatants of mAb-stimulated control cells ranged from 26 to 616 pg/mL with a mean value of 124 pg/mL. The inhibition of IL-12p70 levels was dose-dependent in the 14 subjects tested with a Spearman's r values ranged between -0.72 and -0.99 (p < 0.001).

On the other hand, activating cells with hkSE has significantly induced production of IL-12p70 at Cd doses ranged from 0.5 to 50 ng/mL (Fig 1B); cytokine levels tended to decrease with the increase in Cd levels as previously reported by our group [42]. Considering the mean of the tested subjects (n = 14), the increase in IL-12p70 levels revealed a strong negative correlation with Cd doses from 0.5 to 50 ng/mL (Spearman's r value = -0.72; p <0.01). Control cells activated by hkSE revealed IL-12p70 values ranged between 22 and 413 pg/mL (mean = 127).

Samples of the same blood volunteers were used to evaluate the levels of Th1, Th2 as well as pro-inflammatory cytokine TNF-α, IL-1β and IL-6 following exposure to Cd at low and moderate doses. Results demonstrated that IFN-γ levels increased significantly up to toxic doses, and then declined with the increase of Cd toxicity as recently reported [42]. Supernatants of mAb- or hkSE-activated control cells revealed values of IFN-γ ranged from 50 to 15900 pg/mL (mean = 3310) and from 56 to 2120 pg/mL (mean = 589), respectively.

**Figure 2:**
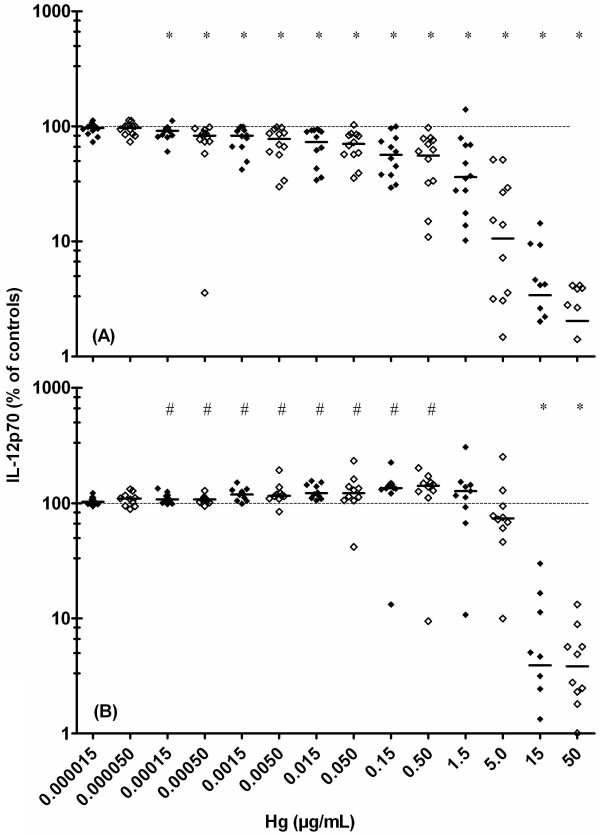
**Levels of IL-12p70 released by human PBMC exposedto HgCl_2 _for 24 hr**. Cells were stimulated either by mAb (anti-CD3/anti-CD28/anti-CD40) (A) or hkSE (B). Data represent the values of 12 and 10 donors in both panels respectively; the horizontal lines represent the medians. Symbols above each plot show whether, cytokine release was significantly stimulated (#) or suppressed (*) compared to the controls (Wilcoxon's Rank Sum test for paired samples; p < 0.05).

Similarly, exposure to Hg at doses ranged from 15 pg to 50 μg/mL significantly decreased IL-12p70 release (Fig. 2), also in a dose-dependent manner (Spearman's r range of -0.64 to -0.97; p < 0.01); additional file 2. In hkSE-stimulated cells, however, the release of IL-12p70 was significantly increased at Hg doses from 15 pg/mL to 0.5 μg/mL, but decreased again with the increase of Hg toxicity. Again, the behavior of IL-12p70 secretion was positively correlated to the levels of IFN-γ previously reported by our group [26].

### Correlation between IL-12 and IFN-γ levels *in vitro*

In both cases of cell activation, testing the relationship between the levels of IL-12p70 and IFN-γ released by cells exposed to either Cd or Hg indicates that they are positively correlated (Table 1). In cases where a significant correlation was evident, correlation test revealed Spearman's r values ranging from 0.63 to 0.97 and from 0.7 to 0.9 under stimulation by mAb or hkSE, respectively. Considering the mean values of the measured cytokines, r values ranged from 0.93 to 1 and from 0.59 to 0.71 have been emerged indicating a strong positive correlation between levels of both cytokines.

**Table 1:** The correlation between IFN-γ and IL-12p70 released by human PBMC.

	***Cadmium acetate***				***Mercuric chloride***			
	
	mAb-stimulation		hkSE-stimulation		mAb-stimulation		hkSE-stimulation	
Donor	r	p	r	p	r	p	r	p
1	0.84	0.0006	0.41	0.1826	0.85	<0.0001	0.72	0.0024
2	0.83	0.001	0.51	0.0893	0.96	<0.0001	0.73	0.0022
3	0.95	<0.0001	0.06	0.8629	0.94	<0.0001	0.80	0.0004
4	0.83	0.001	0.70	0.0114	0.89	<0.0001	0.70	0.0034
5	0.90	<0.0001	0.86	0.0003	0.27	0.3278	0.87	<0.0001
6	0.90	<0.0001	0.72	0.0082	0.96	<0.0001	0.85	<0.0001
7	0.64	0.0261	0.50	0.1006	0.91	<0.0001	0.44	0.0983
8	0.90	<0.0001	0.87	0.0003	0.63	0.0121	0.86	<0.0001
9	0.93	<0.0001	0.76	0.0045	0.16	0.5499	0.26	0.3549
10	0.63	0.0283	0.83	0.001	0.84	<0.0001	0.90	<0.0001
11	0.97	<0.0001			0.23	0.4201		
12	0.94	<0.0001			0.78	0.0007		
13	0.80	0.0016						
14	0.94	<0.0001						

*Mean*	*1*	*<0.0001*	*0.59*	*0.0415*	*0.93*	*<0.0001*	*0.71*	*0.0032*

Human peripheral blood mononuclear cells (PBMC) were cultured for 24 hr in absence or presence of cadmium acetate or mercuric chloride and stimulated either by monoclonal antibodies (mAb: anti-CD3/anti-CD28/anti-CD40) or heat-killed *Salmonella enterica *serovar Enteritidis (hkSE). Cytokine values were evaluated in cell supernatants using ELISA; r represents Spearman correlation values estimated for each sample. The last raw represents the Spearman correlation between the mean values of IFN-γ and IL-12p70 of all investigated donors.

### Serum cytokine profiles of Cd- and Hg-exposed mice

**Figure 3:**
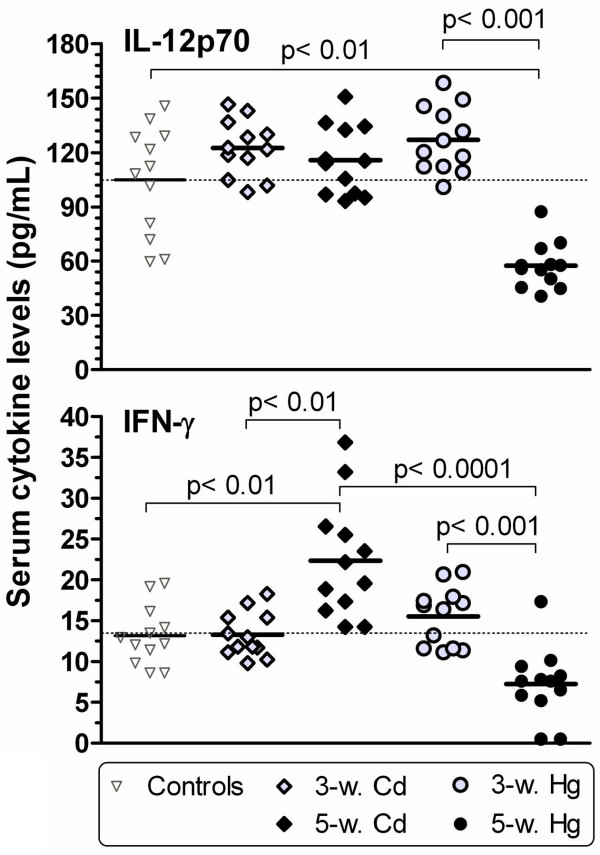
**Serum profiles of IL-12p70 and IFN-γ in BALB/c mice exposed to heavy metals**. Data represent cytokines levels following 3- (3-w) or 5-week (5-w) exposure to cadmium (Cd) acetate or mercuric (Hg) chloride as evaluated by cytometric bead array. The horizontal bars represent the medians of 12 samples. The dotted line represents the control level.

**Figure 4:**
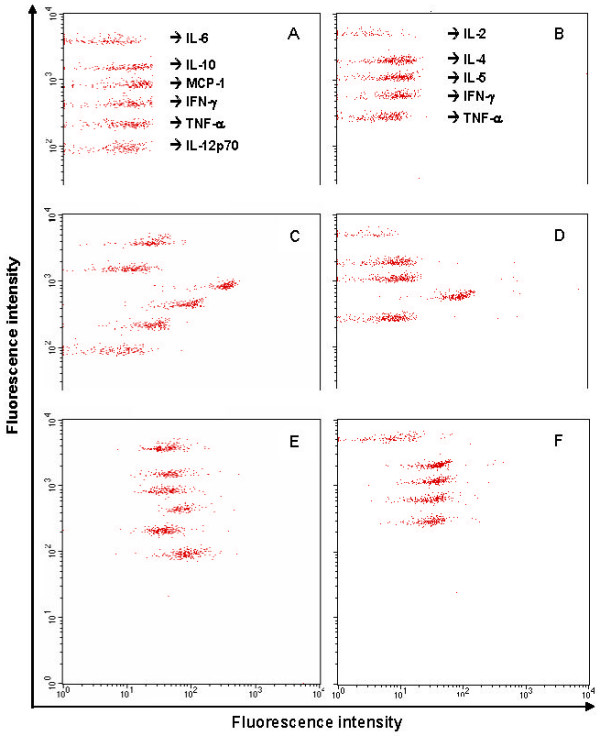
**Serum cytokine levels as evaluated by cytometric bead array (CBA)**. Representative plots show serum cytokine levels of control mice injected every other day for 3 weeks with isotonic saline solution (A) and following 3-week (B) or 5-week (C) exposure to cadmium acetate. Animals were sacrificed and blood samples were collected by heart puncture. Following separation of sera, cytokine content was evaluated by CBA as described in Materials and Methods.

Figure 3 shows serum profiles of IL-12p70 and IFN-γ analyzed by CBA in sera of mice exposed intraperitoneally to Cd acetate or Hg chloride for 3 or 5 weeks. Representative plots of control (PBS-treated) and of Cd-exposed mice are represented in figure 4. The results demonstrate that following exposure to Cd for 5 weeks, the levels of IL-12p70 were positively correlated to the up- or down-regulation of the Th1 cytokine IFN-γ (r = 0.81, p < 0.01), which was in turn negatively correlated to the Th2 cytokines IL-4 (r value = -0.74, p < 0.01) and IL-5 (r value= -0.61, p < 0.01); data not shown. Although the increase in IL-12p70 serum profile of mice exposed for 3 weeks to Cd was not significant in comparison to control mice, exposure to Cd for 5 weeks significantly increased the levels of serum IFN-γ that coincided with the increase in IL-12p70 levels. However, in 3 out of a total of 12 examined mice, where IL-12 release was induced, as in case of 5-week exposure to Cd acetate (Fig. 4C), both of Th1 and Th2 cytokines were also elevated comparable to control mice. In case of Hg-exposure, on the other hand, the decrease in IFN-γ levels, due to the progressive exposure to the metal beyond the first three weeks, was significantly correlated to the decrease in IL-12p70 throughout the same exposure period (r = 0.89, p < 0.01).

### Cytokine gene expression of Cd- and Hg-exposed mice

**Figure 5:**
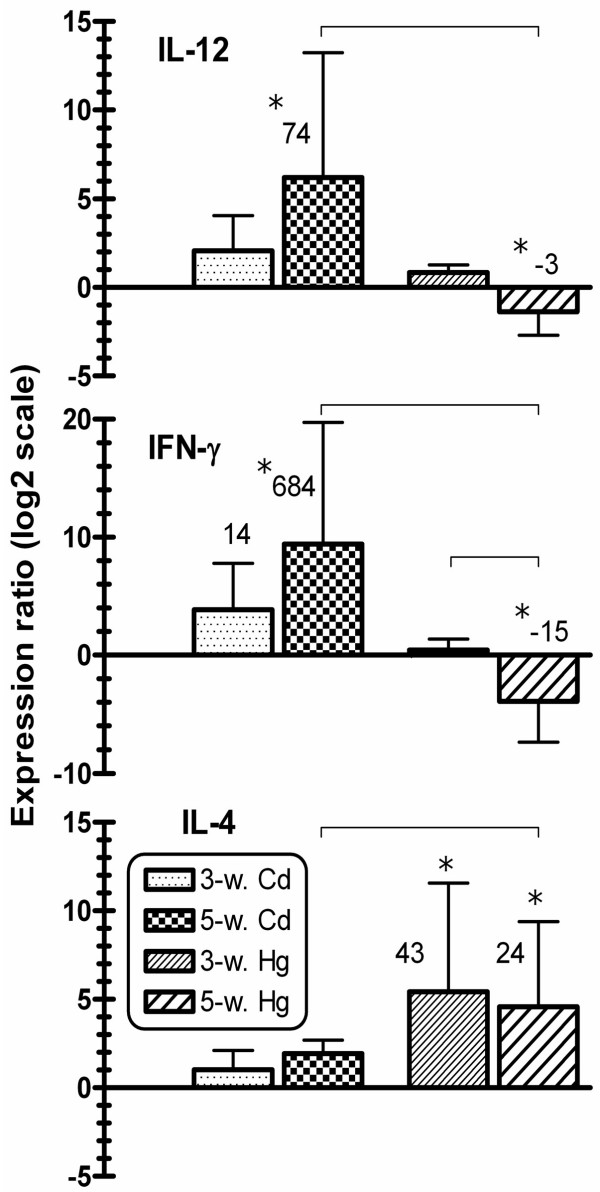
**Relative cytokine mRNA expression in splenocytes of heavy metal-exposed BALB/c mice**. Data represent mRNA relative expression values ± SEM in spleen cells of mice exposed to Cd acetate or Hg chloride for 3 or 5 weeks (3-w, 5-w). Data were analyzed using the REST^© ^software as described in Materials and Methods. Control values were assigned to 1, values of relative gene expression > or < 1 indicate the up- or down-regulation of this gene relative to control mice. The asterisks (*) indicate significance relative to controls as obtained by the pair-wise fixed reallocation randomization test at p < 0.05; the horizontal lines indicate significant differences between the connected groups.

Results of RT-PCR revealed that following exposure of BALB/c mice to salts of Cd or Hg, mRNA expression of IL-12p40, IL-4 and IFN-γ has been significantly modified relative to control mice (Fig. 5). Exposure to Cd for 5 weeks resulted in a significant 74-fold increase in IL-12p40 gene expression, whilst 5-week exposure to Hg decreased its expression 3 folds relative to controls; significant differences between the levels of IL-12p40 in both metals and during both time lapses have been indicated.

As an indicator for both of Th1 and Th2 responses, expression of IFN-γ and IL-4 mRNA was assessed and compared with the serum proteins. In concordance with the increase in IL-12p40 mRNA expression ratios, exposure to Cd resulted in 14- and 684-fold increase in IFN-γ following 3- or 5-week exposition, respectively. Similarly, following 5-week exposure to Cd, the relative expression of IL-12p40 mRNA was positively correlated to that of IFN-γ mRNA (r value = 0.98, p < 0.001).

In case of Hg-exposed mice, on the other hand, 5-week exposition yielded 15-fold decrease in IFN-γ mRNA. The increase in IFN-γ mRNA expression relative to control mice was also correlated to the increase in IL-12p40 mRNA expression. Here, recalling the decrease in IFN-γ mRNA in the 5-week Hg-group relative to the 3-week group, the exposure period constituted a significant factor. Furthermore, it is evident that both metals behave differently. Interesting was the expression of IL-4 mRNA, where a 4-fold increase of this cytokine gene was evident following 5-week exposure to Cd. Exposure to Hg either for 3 or 5 weeks caused 43- or 24-fold increase in IL-4 mRNA expression relative to control mice, respectively. Following 5-week exposure to Hg, IL-12p40 mRNA expression ratios were positively correlated to those of IFN-γ (r value = 0.7, p < 0.001) and negatively correlated to those of IL-4 mRNA (r value = 0.85, p < 0.0001).

## Discussion

To extend our knowledge on heavy metal-elicited immune modulation, the current study was conducted to address the modification of IL-12 profile and its relation to the Th1/Th2-polarized immune responses. Previous reports on this issue indicated that Cd- or Hg-induced immunomodulation was dependent on cell stimulation pathways [26,42]. Moreover, *in vivo *data using BALB/c mice revealed a distinct Th1/Th2 pattern following exposure to sub-lethal doses of Cd acetate or Hg chloride for different periods [43]. These effects were evident even at low concentrations of both metals, at which only insignificant changes in cell vitality were detected, indicating that "cytokines", may serve as more sensitive indicators for heavy metal-induced immune modulation than other parameters such as vitality response. The current data indicates the synergism between IL-12 and IFN-γ and reveals distinct patterns of response to heavy metals depending on the metal ion itself as well as duration of exposure.

In this study, cytokines were quantified by ELISA, CBA, and RT-PCR. In accordance with previous reports [14,26,40], the three methods revealed comparable results indicating their relevance to evaluate the immune response. Comparing the serum cytokine profiles of heavy metal-exposed mice with mRNA expression ratios revealed an overall concordance between them (spearman's r value in all cases ≥ 0.81, p < 0.01). This infers that these metals exert a modulatory effect at the pre-transcriptional stages of cytokine production and indicates the relevance of CBA and RT-PCR to evaluate such modulation. However, evaluating cytokines by the PBA-based assays offers definitive advantages including being quantitative. RT-PCR is quantitative as well [41], but only using other reference house-keeping gene for calibration, which can be even affected by the treatment. Another advantage of the PBA over enzyme immunoassays (EIAs) or ELISA and RT-PCR is that it is performed with a smaller sample volume to measure multiple analytes simultaneously in a single sample [12,14-16,40]. This factor reduces execution time and costs, even adds more precision to the assay and is an advantage when the sample volume is limited as in pediatrics, disease surveillance or in experiments using animal models [12,16,40]. Therefore, and because of the longer processing time and the elaborate sample preparation as well as the costs of RT-PCR techniques, quantifying cytokines using the PBA offers a great ease of performance. In comparison with ELISA, the wide calibration range of the PBA offers a great flexibility [40]. Moreover, analyzing the performance of an established HIV-1 PBA for detection of plasma Abs revealed its advantage in the resolution of weakly reactive samples comparable to two EIAs and Western blot technique [16], the former being able to offer the high throughput of EIA combined with the individual protein testing capacity of the Western blotting.

However, PBA, like other assays, has its limitations. One of the most consistent observations is that, although it is more flexible, user friendly and cost-effective than ELISA, both still detect non-overlapping parameter populations. Another limitation may be the interference with some serum proteins as some samples revealed different results when measured with different titrations. Replacing such *low-sensitive *parameters with more reliable agents and applying sample dilutions may relieve such problems. Here, the use of 20 μL of 1:2 diluted serum samples allowed quantification of cytokines adequately. Taken together, the PBA may be ranked as the most relevant to quantify cytokines in animal experiments and for immune surveillance in heavy metal-exposed subjects. Optimizing the assay may include acquiring a large number of individual bead measurements at low ligand concentrations [13]. This will improve cost benefits and raise the reproducibility of PBA. A recently established analytical workflow [17] may offer the possibility for a subsequent population-level analysis to test hypotheses generated by donor-profile analysis and thereby adding scientific insights to allow the practical distinguishing between different cohorts of the same population using such a promising tool of immune detection.

The results described here are consistent with previous reports and support the reciprocal interplay between Th1 and Th2 immune responses. The early preference of the Th differentiation was believed to depend on the balance between IL-12 (Th1 responses) and IL-4 (Th2 responses) profiles [44]. Infection models of *Listeria*, *Leishmania*, *Schistosoma *and *Toxoplasma *inferred that IL-12 induced Th1 immune responses and inhibited Th2 cells differentiation [45,46]. Similarly, in peripheral type 1 response, IL-12 induced the synthesis of IFN-γ, IL-2, and TNF-α [47]; TNF-α implicated in mediating the effects of IL-12 on NK cells. Furthermore, IL-12 and TNF-α were found to activate IFN-γ-producing NK and T cells [34,48]. Therefore, administration of IL-12 reversed the Th2-dominant heavy metal-induced inhibition of host defense and boosted the resistance of untreated mice marked by elevation of IFN-γ profiles [35]. Similar effects were elucidated in wild-type as well as in IL-12 KO and IL-4 KO BALB/c mice [49,50], where IL-12 was indispensable for the induction of a protective Th1 response against *Salmonella *infection. The possible promotion of IL-12 in Th1 effector function was confirmed by the observation that fully differentiated Th1 cells continue to express functional IL-12 receptor [51]. The loss of the transcription factor GATA-3 expression by developing Th1 cells [52], an augmenting Th2-cell-specific factor [53], constitute an additional evidence for the requirement of a continued IL-12 signaling in the maintenance of a Th1 response.

However, there is evidence that diminishes the role of IL-12 in favoring a Th1-type immune response and indicates that the differentiation into either Th1 or Th2 phenotype does not constitute a simple dichotomy. For example, although *Leishmania major *evokes an IL-4 response in IL-12p40 KO mice [54], the diminished Th1 response observed in STAT4-deficient mice following infection with *L. major *or *Toxoplasma gondii *was not accompanied by a compensatory Th2 response [55]. Furthermore, neither the depletion of IFN-γ in IL-12 KO mice nor the neutralization of IL-12 in IFN-γ R KO mice affected the corona virus-elicited type 1 cytokine pattern [56], and hence IL-12 was proposed not to be essential for the generation of a Th1-polarized response and that viruses may selectively induce IFN-γ production even in the absence of IL-12. Other cytokines such as IFN-α [57] or other host factors or signaling elements, e.g. MyD88 [58], may compensate for the lack of endogenous IL-12 or IFN-γ in determining Th cell differentiation in such viral infections. Recently, it has been found that bacterially-stimulated DC can not produce IL-12 unless the cultures also contain other cells or cytokines such as IFN-γ [59]. Taken together, these data recall the importance of IL-12 for Th1 competence rather than cell priming *per se *[60], i.e. it is involved in the maintenance of an ongoing Th1 response rather than part of the initial immunological decision-making process. In the current study, the finding that production of IL-12 depends on cytokines from previously activated T cells may indicate a direct effect of heavy metals on Th1- or Th2-priming cytokine milieu (e.g. IFN-γ or IL-4). According to the current results inferring the close association between IL-12p70 and the induction of Th1 response, it seems that IL-12 may play a central role in attaining a polarized Th1/Th2 response in heavy metal-exposed individuals even in the absence of pathogenic antigens. This may raise the importance of IL-12 as a target for therapeutic approaches to re-stabilize the immune response in heavy metal-exposed individuals.

## Conclusion

Overall, consistent with previous reports, the results described herein that elucidate the synergism between IL-12 and the Th1 cytokine IFN-γ, which in its turn was shown to be reciprocally regulated by IL-4 (a key Th2-cytokine), may dominate IL-12 as a relevant indicator for heavy metal-induced immune modulation. Moreover, the association of modulating Th cell differentiation and IL-12 levels support the use of this cytokine as a target for therapeutic approaches to re-establish a normal balance that may modify Th1- or Th2-dependent diseases. Finally, measuring cytokines by CBA seems to be the most relevant and cost-effective methodology to evaluate immune modulation.

## Competing interests

The author declares that they have no competing interests.

## Authors' contributions

NYAH is the sole author and is responsible for the entire manuscript.

## Supplementary Material

Additional file 1 **ELISA_IL12_CdAc.** This sheet represents ELISA raw data of IL-12p70 (pg/mL) released by human PBMC following exposure to serial concentrations of cadmium acetate. Click here for file

Additional file 2 **ELISA_IL12_HgCl2.** This sheet represents ELISA raw data of IL-12p70 (pg/mL) released by human PBMC following exposure to serial concentrations of mercuric chloride. Click here for file
